# Supplementary material for: How Well Can Quantum Embedding Method Predict the Reaction Profiles for Hydrogenation of Small Li Clusters?
Source: Nanomaterials (Basel). 2024 Jul 29;14(15):1267. doi: 10.3390/nano14151267 (PMC11313742; doi:10.3390/nano14151267)
Supplement: Supplementary file 1 [file nanomaterials-14-01267-s001.zip › nanomaterials-3103574-supplementary.pdf]

Supplementary Information

How Well Can Quantum Embedding Method Predict the Reaction Profiles for Hydrogenation of Small Li Clusters?

Dominic Alfonso [1,*], Benjamin Avramidis [2,3], Hari P. Paudel [1,2] and Yuhua Duan [1,*]

[1] National Energy Technology Laboratory, U. S. Department of Energy, Pittsburgh, PA 15236, USA
[2] NETL Support Contractor, 626 Cochran Mill Road, Pittsburgh, PA 15236, USA; bavrimidis@pitt.edu (B.A.); hari.paudel@netl.doe.gov (H.P.P.)
[3] Department of Chemistry, University of Pittsburgh, Pittsburgh, PA 15260, USA
* Correspondence: dominic.alfonso@netl.doe.gov (D.A.); yuhua.duan@netl.doe.gov (Y.D.)

REACTION PROFILE COORDINATES (Å)

$Li_2 + H_2$

0

Li -0.238989 -1.070077 -0.049760

Li 2.498442 -1.069539 -0.050301

H 1.130959 3.248352 0.537908

H 1.130888 3.381063 -0.200247

1

Li -0.237676 -0.946930 -0.042502

Li 2.499392 -0.946017 -0.043207

H 1.129745 2.973300 0.528793

H 1.129839 3.122712 -0.205958

2

Li -0.238911 -0.828156 -0.036418

Li 2.500126 -0.828456 -0.036694

H 1.130080 2.702167 0.522024

H 1.130004 2.852667 -0.212288

3

Li -0.241192 -0.729521 -0.031062

Li 2.502546 -0.729531 -0.031233

H 1.130004 2.417640 0.515445

H 1.129942 2.574839 -0.217128

4

Li -0.248846 -0.670829 -0.028560

Li 2.510444 -0.671054 -0.029029

H 1.129886 2.112699 0.510949

H 1.129817 2.283635 -0.218129

5

Li -0.266751 -0.640438 -0.035432

Li 2.525700 -0.640266 -0.036012

H 1.131220 1.800170 0.517195

H 1.131130 1.970034 -0.211452

6

Li -0.281911 -0.510710 -0.042678

Li 2.541297 -0.511719 -0.042570

H 1.130951 1.539989 0.529764

H 1.130963 1.635767 -0.210771

7

Li -0.246597 -0.221652 -0.042581

Li 2.508314 -0.223172 -0.042770

H 1.130005 1.449715 0.536096

H 1.129578 1.424962 -0.216545

8

Li -0.139152 -0.004479 0.002582

Li 2.399663 -0.005170 0.002488

H 1.130397 1.207128 0.580001

H 1.130392 1.292521 -0.350771

9

Li -0.082802 0.186855 0.026138

Li 2.343444 0.186923 0.025684

H 1.130502 1.062148 0.927764

H 1.130149 1.189408 -0.732551

10

Li -0.059646 0.159111 0.027977

Li 2.320249 0.158913 0.027772

H 1.130376 0.767274 1.139258

H 1.130349 0.862915 -1.011686

11

Li -0.035479 0.567340 0.035190

Li 2.295898 0.566591 0.035794

H 1.130271 0.852041 1.315619

H 1.130564 1.362539 -1.071520

12

Li -0.017927 0.748780 0.069639

Li 2.278458 0.747896 0.069266

H 1.130489 0.670366 1.411031

H 1.130245 0.975815 -1.254205

13

Li -0.015743 0.577284 0.054680

Li 2.276467 0.576494 0.054381

H 1.130392 0.496056 1.404102

H 1.130192 0.678406 -1.294084

14

Li -0.015460 0.386657 0.036462
Li 2.276222 0.386267 0.036210
H 1.130439 0.331090 1.388087
H 1.130150 0.425852 -1.316804

15
Li -0.015424 0.192824 0.017681
Li 2.276155 0.192556 0.018050
H 1.130164 0.166984 1.370986
H 1.130498 0.201766 -1.335756

16
Li -0.016207 0.000000 0.000000
Li 2.276760 0.000000 0.000000
H 1.130440 0.000000 1.351365
H 1.130440 0.000000 -1.351365

Li_3 + H_2

0
Li -0.006821 0.826940 0.732104
Li -1.586401 -1.187568 -0.409977
Li 1.579127 -1.187573 -0.401800
H -0.378035 1.927227 2.342216
H 0.392129 1.920774 2.337656

1
Li -0.006896 0.861055 0.557754
Li -1.589798 -1.220365 -0.411153
Li 1.582094 -1.220097 -0.403974
H -0.378065 1.877255 2.196872
H 0.392666 1.869983 2.193463

2
Li -0.007471 0.935295 0.365478
Li -1.586698 -1.236918 -0.377141
Li 1.580199 -1.235164 -0.368157
H -0.378605 1.807488 2.084545
H 0.392574 1.799026 2.080894

3
Li -0.007629 0.984609 0.184754
Li -1.584058 -1.246722 -0.347616
Li 1.578138 -1.243599 -0.339340
H -0.379141 1.738577 1.955162

H 0.392689 1.729148 1.951304

4
Li -0.008719 1.015111 -0.153139
Li -1.578512 -1.270282 -0.329088
Li 1.575133 -1.263546 -0.320157
H -0.380619 1.622397 1.666046
H 0.392717 1.610076 1.661637

5
Li -0.009224 1.016052 -0.313095
Li -1.568877 -1.277825 -0.317110
Li 1.563989 -1.270416 -0.306072
H -0.380231 1.566521 1.517025
H 0.394342 1.557060 1.511315

6
Li -0.009936 1.108704 -0.378367
Li -1.560571 -1.202516 -0.217400
Li 1.547949 -1.197240 -0.203731
H -0.377513 1.458256 1.520968
H 0.400070 1.464046 1.511712

7
Li -0.005607 1.151601 0.075881
Li -1.518751 -1.105119 -0.186508
Li 1.514885 -1.098400 -0.178503
H -0.760877 1.428711 1.547270
H 0.770351 1.413796 1.539188

8
Li -0.025962 1.333230 -0.135077
Li -1.490738 -0.926059 -0.054821
Li 1.518832 -0.886873 -0.030412
H -0.754198 1.165118 1.394445
H 0.752066 1.026535 1.344770
9
Li -0.019186 1.431850 0.118187
Li -1.537074 -0.782008 0.383332
Li 1.559059 -0.759476 0.342475
H -0.977680 1.129139 1.480618
H 0.974880 1.045208 1.443258

10
Li -0.013573 1.435565 -0.093621

Li -1.438474 -0.678209 0.102820
Li 1.470548 -0.693693 0.118518
H -1.134772 0.832669 1.113816
H 1.116272 0.755374 1.064077

11
Li 0.001311 1.482591 0.210216
Li -1.531558 -0.684557 0.151152
Li 1.532493 -0.708604 0.146245
H -1.381705 0.819500 0.988098
H 1.379460 0.787459 0.968096

12
Li 0.002126 1.527893 0.528820
Li -1.553908 -0.749726 0.260182
Li 1.549506 -0.758664 0.255063
H -1.560607 0.877039 0.836177
H 1.562882 0.868387 0.826236

13
Li 0.001491 1.482750 0.453843
Li -1.544094 -0.833522 0.118904
Li 1.540985 -0.837754 0.117504
H -1.604092 0.845334 0.538334
H 1.605710 0.841555 0.534112
14
Li 0.000681 1.432935 0.288884
Li -1.535120 -0.907111 0.011707
Li 1.533739 -0.908801 0.011615
H -1.612855 0.807596 0.251938
H 1.613555, 0.806015 0.250649

15
Li 0.000113 1.398741 0.000000
Li -1.532696 -0.965838 0.000000
Li 1.532453 -0.966123 0.000000
H -1.614487 0.767019 0.000000
H 1.614617 0.766700 0.000000

$Li_4 + H_2$ (Path 1)

0
Li -0.001188 0.902054 0.453030
Li -1.330054 -1.468798 -0.870289
Li 1.292285 -1.482913 -0.875175

Li -0.056791 -3.958241 -1.994248
H -0.359216 2.605744 3.134885
H 0.391064 2.604453 3.127498

1
Li 0.007714 1.036255 0.481817
Li -1.329046 -1.355475 -0.744264
Li 1.290929 -1.360698 -0.787781
Li -0.044228 -3.836131 -1.910289
H -0.373460 2.176193 2.820915
H 0.377418 2.198566 2.827457

2
Li 0.018464 0.992886 0.647742
Li -1.357668 -1.149044 -0.732733
Li 1.315496 -1.154061 -0.782633
Li -0.043000 -3.648349 -1.626634
H -0.377814 1.828031 2.533624
H 0.371319 1.860930 2.540990

3
Li -0.005173 0.544745 0.987302
Li -1.633045 -1.087935 -0.662867
Li 1.585626 -1.087288 -0.702273
Li -0.036555 -3.359784 -1.573533
H -0.367254 1.995175 2.379458
H 0.388390 1.988780 2.372852

4
Li -0.002146 0.422727 0.832393
Li -2.032879 -1.043218 -0.541000
Li 1.987252 -1.034555 -0.606122
Li -0.033419 -3.004718 -1.375498
H -0.379028 1.793270 2.222783
H 0.390959 1.796856 2.215326

5
Li 0.000575 0.071783 0.654663
Li -2.362309 -1.007083 -0.721321
Li 2.318538 -1.011709 -0.792640
Li -0.026874 -2.692025 -1.002248
H -0.382417 1.706726 2.243917
H 0.380308 1.714603 2.241466

6

Li -0.005635 -0.065841 0.285537
Li -2.722197 -1.167046 -0.518630
Li 2.681967 -1.177513 -0.600519
Li -0.030960 -2.350069 -1.127846
H -0.373992 1.823658 2.341815
H 0.380898 1.833760 2.339533

7
Li -0.009930 0.006606 0.579082
Li -2.730272 -1.057739 -0.362413
Li 2.683492 -1.054796 -0.435295
Li -0.031477 -2.131592 -1.076827
H -0.373202 1.571463 2.016304
H 0.391630 1.571185 2.005892

8
Li 0.008064 0.112758 0.261959
Li -2.712097 -0.930758 -0.205161
Li 2.692591 -0.928279 -0.338594
Li -0.027807 -2.163198 -1.056559
H -0.407185 1.148767 1.827323
H 0.366934 1.171610 1.823732

9
Li -0.012724 0.210928 -0.280274
Li -2.683878 -0.996670 -0.102415
Li 2.656058 -1.007962 -0.192452
Li -0.026379 -2.411377 -0.658120
H -0.395246 1.161236 1.317425
H 0.384425 1.170112 1.310591

10
Li -0.020105 0.245524 -0.729972
Li -2.481224 -0.771216 0.276954
Li 2.453009 -0.800532 0.206601
Li -0.036051 -2.445772 -0.619368
H -0.403148 0.888927 0.933964
H 0.410546 0.884476 0.923905

11
Li -0.016904 0.187233 -1.014179
Li -2.046892 -0.561946 0.527747
Li 2.026525 -0.612591 0.484515
Li -0.047092 -2.681807 -0.604884
H -0.590739 0.751073 0.507685

H 0.599266 0.735109 0.496715

12
Li -0.013647 0.540741 -0.627535
Li -1.990021 -0.724320 0.576181
Li 1.971191 -0.769103 0.518607
Li -0.046833 -2.836040 -0.626849
H -1.396024 0.835215 0.345622
H 1.399308 0.801319 0.310713

13
Li -0.014482 0.611812 0.192885
Li -2.269298 -0.781874 0.494862
Li 2.249661 -0.782795 0.415304
Li -0.035898 -2.386892 -1.047498
H -1.655250 0.762750 0.792557
H 1.647164 0.761355 0.733985

14
Li -0.009808 1.151468 0.643937
Li -1.843382 -0.858825 0.320945
Li 1.819273 -0.855777 0.271711
Li -0.028381 -2.800558 -1.079850
H -1.703651 0.787972 0.783836
H 1.687660 0.790426 0.739395

15
Li -0.000605 1.461445 0.544592
Li -1.533137 -0.862604 0.116532
Li 1.501043 -0.877860 0.089446
Li -0.035099 -3.352529 -0.615869
H -1.605146 0.833278 0.469478
H 1.595865 0.816752 0.442969

16
Li 0.004776 1.464800 0.000103
Li -1.521762 -0.914903 -0.003807
Li 1.483418 -0.942162 -0.004333
Li -0.039724 -3.537311 -0.038031
H -1.595198 0.820205 0.003549
H 1.593491 0.790972 0.003218

$Li_4 + H_2$ (Path 2)

0

Li -0.046198 1.710357 -0.213665
Li -1.315567 -1.029278 -0.418066
Li 1.314594 -0.998435 -0.424949
Li 0.042226 -3.741115 -0.573967
H -0.240295 -0.975222 5.966313
H 0.019341 -1.677907 5.963424

1
Li -0.046189 1.710366 -0.213676
Li -1.315569 -1.029295 -0.418041
Li 1.314593 -0.998416 -0.424924
Li 0.042219 -3.741133 -0.573977
H -0.240412 -0.974877 5.591329
H 0.019458 -1.678246 5.588442

2
Li -0.046191 1.710366 -0.213673
Li -1.315571 -1.029295 -0.418030
Li 1.314595 -0.998418 -0.424915
Li 0.042221 -3.741132 -0.573974
H -0.240409 -0.974852 5.216327
H 0.019455 -1.678269 5.213443

3
Li -0.046194 1.710365 -0.213668
Li -1.315574 -1.029295 -0.418017
Li 1.314598 -0.998421 -0.424904
Li 0.042224 -3.741131 -0.573970
H -0.240403 -0.974812 4.841324
H 0.019449 -1.678307 4.838445

4
Li -0.046199 1.710362 -0.213662
Li -1.315576 -1.029296 -0.418002
Li 1.314601 -0.998426 -0.424892
Li 0.042229 -3.741126 -0.573966
H -0.240392 -0.974745 4.466323
H 0.019438 -1.678370 4.463446

5
Li -0.046208 1.710355 -0.213658
Li -1.315579 -1.029297 -0.417991
Li 1.314605 -0.998434 -0.424885
Li 0.042237 -3.741117 -0.573969
H -0.240375 -0.974628 4.091325

H 0.019421 -1.678479 4.088444

6
Li -0.046224 1.710341 -0.213662
Li -1.315580 -1.029300 -0.417995
Li 1.314609 -0.998446 -0.424896
Li 0.042251 -3.741102 -0.573991
H -0.240348 -0.974419 3.716336
H 0.019391 -1.678674 3.713433

7
Li -0.046250 1.710316 -0.213687
Li -1.315575 -1.029308 -0.418037
Li 1.314616 -0.998467 -0.424951
Li 0.042277 -3.741077 -0.574060
H -0.240308 -0.974036 3.341368
H 0.019341 -1.679028 3.338401

8
Li -0.046246 1.710311 -0.213604
Li -1.315554 -1.029204 -0.418137
Li 1.314594 -0.998535 -0.425030
Li 0.042258 -3.741029 -0.573909
H -0.240903 -0.973601 2.966339
H 0.019950 -1.679541 2.963430

9
Li -0.064370 1.741136 -0.297783
Li -1.312867 -1.011902 -0.276561
Li 1.313235 -0.956092 -0.309111
Li 0.054987 -3.696564 -0.538898
H -0.111919 -0.798254 2.655898
H -0.089984 -1.553728 2.634793

10
Li -0.077031 1.745198 -0.383375
Li -1.293960 -0.989531 -0.075162
Li 1.309877 -0.919620 -0.159441
Li 0.062652 -3.643280 -0.491223
H -0.108528 -0.666068 2.329783
H -0.091117 -1.429150 2.279455

11
Li -0.082590 1.694373 -0.433350
Li -1.241996 -0.975201 0.198176

Li 1.283870 -0.904227 0.069355
Li 0.064415 -3.570401 -0.462203
H -0.120170 -0.581907 1.984202
H -0.095119 -1.375384 1.956519

12
Li -0.096945 1.542958 -0.428244
Li -1.265973 -0.968459 0.353694
Li 1.193154 -0.894191 0.404008
Li 0.051025 -3.415618 -0.387203
H -0.048636 -0.499301 1.654101
H -0.021566 -1.400964 1.640133

13
Li -0.095102 1.465663 -0.350674
Li -1.215303 -0.950139 0.424414
Li 1.151139 -0.874414 0.453131
Li 0.051170 -3.301588 -0.235062
H -0.067967 -0.067378 1.440939
H -0.009706 -1.815403 1.412578

14
Li -0.084212 1.415640 0.053184
Li -1.196519 -1.045992 0.584292
Li 1.125830 -0.992175 0.619484
Li 0.038869 -3.394052 -0.159149
H -0.071922 0.138925 1.374004
H -0.017772 -2.246604 1.247261

15
Li -0.089321 1.125451 -0.475740
Li -1.194946 -0.975143 0.812023
Li 1.124671 -0.893307 0.841884
Li 0.052811 -3.023461 -0.431877
H -0.088705 0.410258 1.226295
H 0.006856 -2.270464 1.255108

16
Li -0.126501 1.472502 -0.654561
Li -1.186914 -0.784228 0.438402
Li 1.124620 -0.716802 0.438342
Li 0.076387 -3.151933 -0.186192
H -0.025745 0.552024 0.934693
H -0.013871 -1.932390 1.204467

17

Li -0.698918 0.424840 -0.459963

Li -0.973699 -1.473501 1.477839

Li 1.200234 -0.808341 1.066581

Li 0.012445 -2.315905 -1.205798

H -0.295176 0.255586 1.335571

H 0.544816 -2.340056 1.636304

18

Li -0.098013 0.079090 0.090651

Li -0.862098 -1.022889 1.972993

Li 1.608213 -1.958356 0.625491

Li -0.103900 -2.675338 -1.562339

H -1.637259 0.134581 0.948743

H 0.887017 -0.690543 1.652620

19

Li 0.040670 0.732056 1.000834

Li -0.960230 -1.429278 1.179035

Li 1.576647 -2.008846 0.582340

Li -0.201845 -3.316258 -1.258514

H -1.597484 0.222714 1.011750

H 0.923713 -0.697385 1.571719

20

Li 0.040902 0.516657 1.628151

Li -1.547294 -1.409505 0.310022

Li 1.431051 -1.654230 0.468032

Li -0.161634 -3.707323 -1.033949

H -1.571147 0.076327 1.204872

H 1.594033 -0.189688 1.382414

21

Li 0.008202 1.049157 0.852630

Li -1.530470 -1.220046 0.167260

Li 1.462078 -1.279672 0.189135

Li -0.078910 -3.747528 -0.548440

H -1.593822 0.443986 0.652914

H 1.587312 0.380001 0.677255
